# Tumor Suppressor WWOX and p53 Alterations and Drug Resistance in Glioblastomas

**DOI:** 10.3389/fonc.2013.00043

**Published:** 2013-03-04

**Authors:** Ming-Fu Chiang, Pei-Yi Chou, Wan-Jen Wang, Chun-I Sze, Nan-Shan Chang

**Affiliations:** ^1^Department of Neurosurgery, Mackay Memorial HospitalTaipei, Taiwan; ^2^Graduate Institute of Injury Prevention and Control, Taipei Medical UniversityTaipei, Taiwan; ^3^Institute of Molecular Medicine, National Cheng Kung UniversityTainan, Taiwan; ^4^Department of Cell Biology and Anatomy, Nation Cheng Kung UniversityTainan, Taiwan; ^5^Department of Pathology, Nation Cheng Kung UniversityTainan, Taiwan; ^6^Advanced Optoelectronic Technology Center, National Cheng Kung UniversityTainan, Taiwan; ^7^Department of Neuroscience and Physiology, SUNY Upstate Medical UniversitySyracuse, NY, USA; ^8^Department of Neurochemistry, New York State Institute for Basic Research in Developmental DisabilitiesStaten Island, NY, USA

**Keywords:** tumor suppressor, p53, WWOX, glioblastoma multiforme, temozolomide resistance

## Abstract

Tumor suppressor p53 are frequently mutated in glioblastomas (GBMs) and appears to contribute, in part, to resistance to temozolomide (TMZ) and therapeutic drugs. WW domain-containing oxidoreductase WWOX (FOR or WOX1) is a proapoptotic protein and is considered as a tumor suppressor. Loss of *WWOX* gene expression is frequently seen in malignant cancer cells due to promoter hypermethylation, genetic alterations, and translational blockade. Intriguingly, ectopic expression of wild type WWOX preferentially induces apoptosis in human glioblastoma cells harboring mutant p53. WWOX is known to physically bind and stabilize wild type p53. Here, we provide an overview for the updated knowledge in p53 and WWOX, and postulate potential scenarios that wild type and mutant p53, or isoforms, modulate the apoptotic function of WWOX. We propose that triggering WWOX activation by therapeutic drugs under p53 functional deficiency is needed to overcome TMZ resistance and induce GBM cell death.

## Mechanisms of Resistance to Temozolomide in Glioblastomas

Glioblastoma multiforme afflicts 12,500 new patients in the U.S. annually (Friedman et al., 2000; Stupp et al., 2009; Silber et al., 2012). Glioblastoma (GBM) is highly lethal, and the average survival expectancy is 14.6 months, and the overall 5-year survival rate for GBM is only 9.8% (Friedman et al., 2000; Stupp et al., 2009). High levels of resistance to current therapeutic modalities and cancer relapse are frequently seen in patients (Haar et al., 2012; Happold et al., 2012). The current standard therapy for GBM mainly includes maximum debulking surgery, radiation, and treatment with the monofunctional alkylating agent temozolomide (TMZ) (Friedman et al., 2000; Nishikawa, 2010). Multiple mechanisms are involved in the TMZ resistance, which may include cancer stem cells, microRNAs, drug efflux, DNA damage repair, tumor cells under hypoxia, histone deacetylation, epithelial-mesenchymal transition, STAT3 kinase, and many others (Haar et al., 2012; Happold et al., 2012; Johannessen and Bjerkvig, 2012; Kitange et al., 2012; Kohsaka et al., 2012; Zhang et al., 2012b).

Temozolomide induces generation of DNA lesions, including O6-methylguanine, N3-methyladenine, and N7-methylguanine (Goellner et al., 2011; Zhang et al., 2012b). The O6-methylguanine lesion is known to trigger autophagy, rather than apoptosis, to cause cell death (Kanzawa et al., 2003). Also, inhibition of antiapoptotic Bcl-2 by a pan-Bcl-2 inhibitor (−)-gossypol leads to autophagic death in gliomas and enhances the action of TMZ (Voss et al., 2010). However, a recent study demonstrated that TMZ-induced autophagy is pro-survival, and may block the eventual apoptosis in GBM cells (Knizhnik et al., 2013). Also, *MAPO2* (*C1orf201*) gene participates in the O6-methylguanine lesion-induced apoptosis (Fujikane et al., 2012). *MAPO2* gene encodes a novel 37-kDa protein. It is not determined whether this gene is involved in autophagy.

The O6-methylguanine lesion is a substrate for direct repair by O6-methylguanine-DNA methyltransferase (MGMT) (Pollack et al., 2006; Hegi et al., 2008; Fukushima et al., 2009; Zhang et al., 2012b). Without MGMT repair, O6-methylguanine initiates activation of mismatch repair-deficient (MMR) proteins or Rad3-related protein kinase that ultimately leads to apoptotic cell death (Caporali et al., 2004; Wang and Edelmann, 2006; Roos et al., 2007). High expression of MGMT or loss of MMR contributes significantly to TMZ resistance in many clinical cases (Pollack et al., 2006; Hegi et al., 2008; Sarkaria et al., 2008). The initiation of apoptotic signaling fails in the absence of the MMR system.

Sensitivity to TMZ is significantly associated with the methylation status of *MGMT* gene promoter in cells committed to differentiation (Villalva et al., 2012). An increase in *MGMT* gene promoter methylation, which blocks MGMT protein expression, prolongs cancer patient survival. Intriguingly, overexpressed microRNA-21 reduces Bax/Bcl-2 ratio and caspase-3 activity, thereby blocking TMZ-induced apoptosis (Shi et al., 2010). MicroRNA-21 is considered as a pro-survival factor for cancer cells (Li et al., 2012). Integrins play a role in the resistance of advanced cancers to radiotherapy and chemotherapy. α5β1 integrin negatively regulates p53 signaling, and the event induces glioma cell resistance to TMZ (Janouskova et al., 2012). α5β1 integrin is considered as a therapeutic target for high-grade brain tumors. The base excision repair enzyme alkylpurine-DNA-*N*-glycosylase (APNG), which repairs the cytotoxic lesions N3-methyladenine and N7-methylguanine, also participates in the TMZ resistance (Agnihotri et al., 2012). Upregulation of mitochondrial respiratory chain coupling to suppress the production of reactive oxygen species (ROS) regulated by cytochrome c oxidase contributes in part to TMZ resistance in gliomas (Oliva et al., 2011).

Reversal of TMZ resistance may be achieved by MGMT pseudosubstrates, O6-benzylguanine and lomeguatrib to sensitize tumors to TMZ (Zhang et al., 2012b). Methoxyamine-blocker of base excision repair contributes significantly to TMZ cytotoxicity particularly when O6-methylguanine adducts are repaired or tolerated (Goellner et al., 2011; Zhang et al., 2012b). Dual targeting of base excision repair and NAD(+) biosynthesis may reverse TMZ resistance in patients with resistant and recurrent GBM (Goellner et al., 2011). Interferon-β (IFN-β), levetiracetam (LEV), resveratrol, and valproic acid (VAP) increase the sensitivity of TMZ through MGMT-dependent or -independent mechanisms (Nakada et al., 2012). Resveratrol, a natural polyphenol, reverses TMZ resistance via an NF-κB-dependent mechanism (Huang et al., 2012). STAT3 inhibitor or STAT3 knockdown potentiates TMZ efficacy in resistant GBM cell lines (Kohsaka et al., 2012). Intratumoral hypoxia is common in GBMs and may be associated with the development of TMZ resistance. Induced hyperoxia can be utilized to reverse TMZ resistance in GBMs (Sun et al., 2012). Cancer stem cells are probably the key to failure in TMZ treatment. The concept of cancer stem cell survival from treatment with TMZ and other chemotherapeutic drugs has been more complicated than previously thought (Beier et al., 2011; Chen et al., 2012). CD133-positive cancer stem cells are expressed in both normal stem cells and cancer stem cells (Donovan and Pilkington, 2012). However, the role of CD133 as a marker for glioma cancer stem cells relative to its biological function has yet to be established.

## WW Domain-Containing Oxidoreductase WWOX

**Figure 1:**
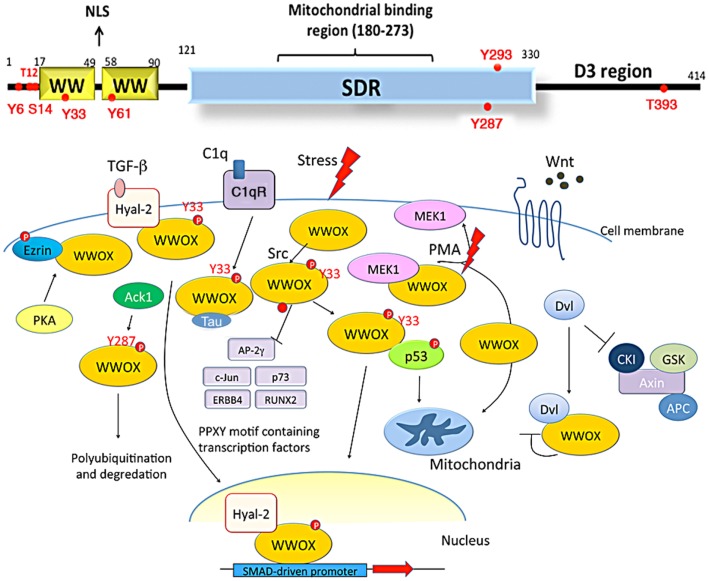
**Schematic diagrams of WWOX structure and its role in signaling**. WWOX contains two *N*-terminal WW domains, a *C*-terminal short-chain alcohol dehydrogenase/reductase (SDR) domain, and a D3 region. A nuclear localization signal (NLS) is in between the WW domains (Chang et al., 2003, 2007; Del Mare et al., 2011; Salah et al., 2012; Su et al., 2012; reviews). The SDR domain possesses a mitochondria-binding region. There are two conserved tyrosine phosphorylation sites, Tyr33 and Tyr287. Other phosphorylation sites predicted by NetPhos 2.0 Server are Tyr6, Thr12, Ser14, Tyr61, Tyr293, and Thr3B3, respectively. Upon stress stimulation, WWOX is phosphorylated in Tyr33 and binds p53. The WWOX/p53 complex then translocates to mitochondria or nucleus to induce apoptosis. WWOX also binds PPxY motif-containing transcription factors, such as RUNX2, c-Jun, and ERBB4, via its first WW domain, and the binding blocks the activity of these proteins by retaining them in the cytoplasm. WWOX binds Tau via its SDR domain. WWOX participates in the Wnt/β-catenin signal pathway by binding Dvl and inhibiting its nuclear import. Phorbol ester stimulates the dissociation of MEK/WWOX complex in Jurkat T cell, and the released WWOX translocates to the mitochondria for causing cell death. WWOX is recruited to the membrane area by association with Hyal-2 and phospho-Ezrin. Hyal-2 is an alternative receptor for TGF-β1. In response to TGF-β1, WWOX binds Hyal-2 and forms a complex, followed by relocating to the nucleus and enhancing the SMAD-driven promoter activity. Ack1, activated Cdc42-associated kinase 1; Hyal-2, hyaluronoglucosaminidase 2; CKI, casein kinase; GSK, glycogen synthase kinase; PKA, protein kinase A.

Recently, tumor suppressors p53 and WWOX were shown to regulate the apoptosis of glioblastoma cells (Chiang et al., 2012). WW domain-containing oxidoreductase, known as WWOX, FOR, or WOX1, is encoded by human or mouse *WWOX*/*Wwox* gene. This gene is located in chromosome 16q23.3–24.1, an area known as the common fragile site *FRA16D*. The full-length WWOX protein is composed of two *N*-terminal WW domains, a *C*-terminal short-chain alcohol dehydrogenase/reductase (SDR) domain, and a proapoptotic *C*-terminal tail D3 (Chang et al., 2001, 2007, 2010; Aqeilan et al., 2004, 2007; Hong et al., 2007; Salah et al., 2012) (Figure 1). WWOX may act as an alternative receptor for sex steroid hormones, since its SDR domain possesses an NSYK motif capable of interacting with androgen and estrogen (Chang et al., 2005a; Su et al., 2012).

Expression of WWOX is either altered or lost from epigenetic modification in multiple malignant cancers, such as non-small cell lung carcinoma (Donati et al., 2007), hematopoietic malignancies (Ishii and Furukawa, 2004), gastric carcinoma (Aqeilan et al., 2004), pancreatic carcinoma (Kuroki et al., 2004), breast carcinoma (Guler et al., 2004; Aqeilan et al., 2007), and glioblastoma multiforme (Kosla et al., 2011). Restoration of *WWOX* gene prevents the growth of lung cancer (Fabbri et al., 2005), pancreatic cancer (Nakayama et al., 2008), and prostate cancer (Hong et al., 2009).

The first WW domain of WWOX may interact with proteins possessing a PPxY motif(s) such as AP-2γ, p73, ErbB4, Ezrin, SIMPLE, c-Jun, RUNX4, and many others (Chang et al., 2007; Salah et al., 2012; Su et al., 2012; Figure 1). Transiently overexpressed WWOX binds transcription factors AP-2, p73, and c-Jun and block their nuclear relocation *in vitro*, which suppresses cancer cell survival (Salah et al., 2012). In stark contrast, Wwox co-migrates with proapoptotic and pro-survival transcription factors to the nuclei of neurons upon sciatic nerve axotomy in rats (Li et al., 2009). No blocking of translocation of transcription factors to the nuclei by Wwox was observed.

Under stress conditions, WWOX is activated via phosphorylation at Tyr33, and binds proteins independently of the PPxY motif (Chang et al., 2001, 2003, 2005a,b, 2007, 2010). Activated WWOX physically interacts with serine 46-phosphorylated p53 (Figure 1), which stabilizes p53 and its apoptotic function (Chang et al., 2005b). Also, WWOX binds Disheveled proteins (Dvl), which are key components in Wnt/β-catenin signaling pathway (Figure 1). No PPxY motif is in Dvl. Transiently overexpressed WWOX sequesters Dvl-2 in the cytoplasm and thereby blocks Dvl-2-mediated TCF transcriptional activity (Bouteille et al., 2009).

Overexpressed WWOX induces apoptosis and inhibits proliferation of human hepatic carcinoma cells (Hu et al., 2012) and many cancer cell types (Chang et al., 2007, 2010; Salah et al., 2012; Su et al., 2012). WWOX enhances the cytotoxic function of tumor necrosis factor by down-regulating apoptosis inhibitor Bcl-2 and Bcl-xL and up-regulating apoptotic p53 (Chang et al., 2001). Also, WWOX mediates cell death synergistically with p53. Upon exposure to chemicals or environmental stress, such as UV irradiation and chemotherapeutic drugs, WWOX undergoes phosphorylation in Tyr33 and probably others sites, followed by relocating to mitochondria or nuclei for inducing apoptosis (Chang et al., 2007, 2010).

WWOX binds MEK in Jurkat T cells (Lin et al., 2011). Phorbol myristate acetate (PMA) dissociates the MEK/WWOX complex and induces WWOX to translocate to the mitochondria to induce apoptosis, whereas MEK relocates to the lipid raft. Inhibition of MEK activity increases TMZ-induced suppression of cancer cell growth (Holt et al., 2012). Ectopic expression of WWOX in A549 cells induces procaspase-3 and procaspase-9 activation and induces cytochrome C releasing from the mitochondria (Zhang et al., 2012a). Complement C1q induces ectopic WWOX phosphorylation in Try33 and leads to cell apoptosis independently of the classical complement activation pathway (Hong et al., 2009). A portion of WWOX is anchored in the membrane/cytoskeleton area via binding with hyaluronidase Hyal-2 (Hsu et al., 2009) and Phospho-Ezrin (Jin et al., 2006). Transforming growth factor β1 binds Hyal-2 as a cognate receptor to signal the formation of the WWOX/Hyal-2/Smad4 complex to relocate to the nucleus for enhancing the SMAD-driven promoter activity (Hsu et al., 2009).

## p53 and Functions

**Figure 2:**
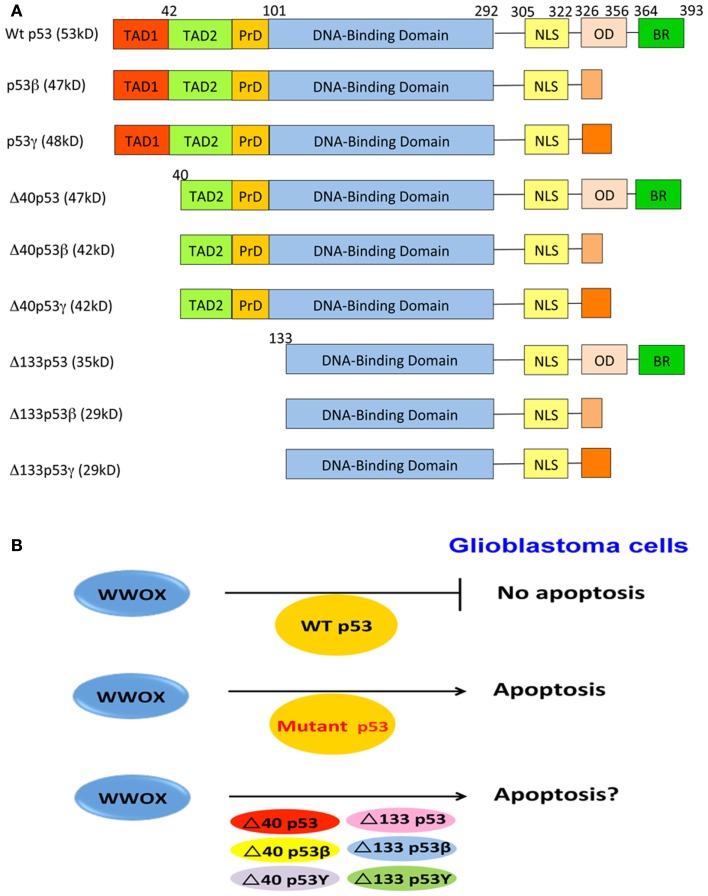
**p53 and WWOX in GBM cell apoptosis**. **(A)** Full-length p53 possesses two *N*-terminal transactivation acidic domains, a proline-rich domain, a central DNA-binding region, and a *C*-terminal domain, containing a nuclear localization signal, an oligomerization domain, and a basic region. *N*-terminal deletion mutants of p53 are also shown. **(B)** Three likely scenarios are proposed for WWOX and p53 to regulate GBM cell death. First, ectopic WWOX fails to induce apoptosis of GBM cells expressing endogenous wild type p53. It appears that ectopic WWOX binds and functionally antagonizes p53, and both proteins nullify each other’s function in inducing apoptosis. Second, no physical interactions between mutant p53 proteins and WWOX are expected in GBM cells. Thus, ectopic WWOX induce apoptosis in mutant p53-expressing cells. Finally, whether WWOX binds p53 isoforms is unknown. Also, whether ectopic WWOX induction of apoptosis in GBM cells expressing p53 isoforms remains to be established. TAD, transactivation domain; PrD, proline domain; NLS, nuclear localization signal; OD, oligomerization domain; BR, basic region.

Activated p53 mediates apoptosis, cell cycle arrest, senescence, DNA repair, or metabolism (Lane and Levine, 2010). The primary structures of p53 and its isoforms are depicted (Figure 2). p53 induces cell cycle arrest by transactivating genes such as cyclin-dependent kinase inhibitor p21, or microRNA miR34. p53 induces apoptosis by transactivating proapoptotic genes such as *BAX*, *PUMA*, *SCOTIN*, and *FAS*, and inhibiting the antiapoptotic gene *BCL-2* (Lane and Levine, 2010). p53 triggers pro-survival or cell death response, depending upon cell types, the intensity of the stress signal, and the extent of cellular damage (Menendez et al., 2009). Also, p53 plays a role in controlling cell motility via regulating the expression of smooth muscle α-actin (Comer et al., 1998), collagens IIα1 and VIα1 (Sun et al., 1999), and many others.

## p53 Isoforms

At least nine isoforms of p53 have been identified due to alternative mRNA splicing, multiple gene promoters, and alternative initiation sites of translation (Ghosh et al., 2004; Ray et al., 2006; Figure 2). In cancers, aberrant expression of p53 isoforms occurs frequently (Bourdon et al., 2005; Bourdon, 2007). Full-length p53, Δ133p53, and p53β are localized mainly in the nucleus, and only few of them are in cytoplasm. p53γ and Δ133p53β are localized in the nucleus and cytoplasm. Δ133p53γ is localized in the cytoplasm (Bourdon et al., 2005). Co-transfection of p53 and p53β increases p53-mediated apoptosis, whereas co-expression of p53 with Δ133p53 suppresses p53-mediated apoptosis (Bourdon et al., 2005). Δ133p53 differentially regulates gene expression in p53-dependent and -independent manners (Aoubala et al., 2011). Co-expression of Δ133p53β or Δ133p53γ with p53 does not affect p53 transcriptional activity on *p21* and *Bax* promoters, as well as apoptosis.

## p53 and WWOX Alterations in GBMs

p53 mutants are considered as unfavorable factors for the effectiveness in radiotherapy and TMZ treatment in glioma cells (Gjerset et al., 1995; Hirose et al., 2001; Squatrito et al., 2010; Blough et al., 2011). Loss of functional p53 confers sensitivity to TMZ in glioma cells, whereas wild type p53 increases the TMZ resistance (Blough et al., 2011). p53 mutants reduce TMZ sensitivity in gliomas (Blough et al., 2011).

Altered WWOX expression is shown in GBMs, in which downregulation of WWOX is associated with loss of heterozygosity and promoter methylation (Kosla et al., 2011). Recently, we demonstrated that overexpressed WWOX induces apoptosis of glioblastoma U373-MG cells harboring mutant p53 by causing hypoploidy and DNA fragmentation (Chiang et al., 2012). However, ectopic WWOX has no effect with U87-MG expressing wild type p53. Unlike TMZ, WWOX induces apoptosis of U373-MG cells via a mitochondria-independent and caspase-3-independent pathway (Chiang et al., 2012). While the underlying mechanisms are unknown, it is reasonable to assume that the survival of human glioblastoma cells depends upon interactions between the gain-of-function of p53 mutants and WWOX. Activated WWOX binds wild type p53 with Ser46 phosphorylation (Chang et al., 2005b). UV irradiation enhances the binding interactions. Despite the presence of Ser46 in Δ40p53, binding of this protein with WWOX remains to be determined. We postulate that in GBM cells, both wild type p53 and ectopic WWOX proteins appear to have a functional antagonism, thereby nullifying each other’s function in inducing apoptosis (Figure 2). Mutant p53 proteins cannot bind ectopic WWOX in GBM cells, and WWOX is able to induce apoptosis. Whether WWOX causes apoptosis in GBM expressing p53 isoforms is unknown and remains to be established.

## Perspectives

Whether WWOX affects TMZ sensitivity has never been determined. Binding proteins for WWOX and/or p53 are likely to affect apoptosis and TMZ sensitivity in GBM cells. WWOX binds MEK, and that PMA dissociates this complex for causing apoptosis of T leukemia cells (Lin et al., 2011). Thus, appropriate chemicals, which break apart the WWOX/MEK complex, are expected to cause GBM cell death. Preliminary studies from our screening of chemicals have selected certain small molecules that induce apoptosis of many types of cancer cells (Lu et al., unpublished). Indeed, specific inhibition of MEK by selumetinib enhances TMZ-induced cancer cell death *in vivo* (Holt et al., 2012). Serum factors can be utilized for blocking cancer growth. For example, serum complement C1q induces apoptosis of prostate cancer and neuroblastomas cells without participation of downstream proteins in the classical activation pathway (Hong et al., 2009). In this event, ectopic WWOX is activated for inducing apoptosis.

Failure of ectopic WWOX in inducing apoptosis of glioma cells possessing wild type p53 is unusual (Chiang et al., 2012). In most cases, we have shown that p53 functionally interacts with WWOX, and both proteins induce apoptosis in a synergistic manner (Chang et al., 2001, 2003, 2005a,b, 2007, 2010, 2012; Su et al., 2012). A likely scenario is that p53-binding proteins, which are present in GBMs, may interfere with the apoptotic function of WWOX and p53. Functional antagonism among tumor suppressors has never been documented in the literature. However, it is not surprising to find that many tumor suppressor proteins, e.g., p53, WWOX, Smad4, PTEN, PP2A, and etc., are significantly upregulated during the early stage of cancer progression (Lai et al., 2005; Chang et al., unpublished). Do these proteins act synergistically in blocking cancer progression but lose control eventually? Or, do they counteract each other’s function, thus allowing cancer growth? Whether endogenous WWOX counteracts with the function of endogenous p53 is unknown and remains to be established.

One of the unique characteristics for malignant gliomas is their diffuse infiltration into distant brain tissue. Signal pathways, involving PI3K, Akt, mTOR, NF-κB, and autophagy, are believed to confer these migrating cells resistant to apoptotic death (Lefranc et al., 2005). Glioma cells possess CD44 as a receptor for interacting with brain matrix hyaluronan (Murai et al., 2004; Yoshida et al., 2012), and secrete hyaluronidases and metalloproteinases to facilitate their migration (Delpech et al., 2002; Junker et al., 2003; Hagemann et al., 2012). Also, lack of WWOX expression is expected to enhance cell migration. For example, loss of WWOX facilitates migration of ovarian cancer and osteosarcoma cells (Gourley et al., 2009; Del Mare et al., 2011). Ectopic expression of TIAF1 (TGF-β-induced antiapoptotic factor), p53, and WWOX suppresses anchorage-independent growth and cell migration and causes apoptosis in cancer cells (Chang et al., 2012). We believe that glioma cells, with stem cell-like properties, migrate individually rather than collectively. This assumption is based upon our observations that mouse *Wwox* gene knockout cells (e.g., embryonic fibroblasts) migrate individually and aggressively. In contrast, wild type cells migrate collectively (Chou et al., unpublished). While a portion of WWOX is anchored on the cell membrane/cytoskeleton, WWOX-negative cells lose recognition by parental WWOX-positive cells, and this increases the mobility of WWOX-negative cells to move away from the WWOX-positive cells. Conceivably, WWOX-negative glioma cells migrate individually and turn away from WWOX-expressing brain cells to low WWOX expression areas. Apparently, the migratory behavior of WWOX-negative glioma cells may account for the diffuse invasion into distant brain tissue (Chou et al., unpublished).

Metabolic alterations have been shown in *Wwox* knockout mice, including postnatal lethality, bone metabolism defects, ataxia, steroidogenesis, and generation of osteosarcomas (Del Mare et al., 2011; Salah et al., 2012). In a *Drosophila* model, *Wwox* is shown to participate in pathways involving aerobic metabolism and oxidative stress for generation of ROS (O’Keefe et al., 2011). Under UV stress, functional *Wwox* gene expression induces ROS production in *Drosophila*. Cancer cells are known to overly utilize glycolysis for growth advantage – the so-called Warburg (Moncada et al., 2012). Conceivably, WWOX is likely to override glucose consumption in cancer cells and exerts generation of ROS to curb the cancer cell growth and invasion.

In summary, in this perspective article we have discussed the potential role of tumor suppressors p53 and WWOX in regulating TMZ sensitivity in GBM cells. We have shown the role of WWOX in controlling cell migration and metabolic alterations, and discussed the effects of WWOX deficiency and TMZ resistance in cancer cells.

## Authors Contribution

Ming-Fu Chiang and Chun-I Sze wrote the sections dealing with TMZ; Pei-Yi Chou wrote the review sections for p53 and WWOX and designed graphs; Wan-Jen Wang managed citations; Nan-Shan Chang conceived ideas, wrote and thoroughly revised the manuscript, and discussed with coauthors.

## Conflict of Interest Statement

The authors declare that the research was conducted in the absence of any commercial or financial relationships that could be construed as a potential conflict of interest.
